# Stable duplex-linked antisense targeting miR-148a inhibits breast cancer cell proliferation

**DOI:** 10.1038/s41598-021-90972-3

**Published:** 2021-06-01

**Authors:** Sho Okumura, Yu Hirano, Yasuo Komatsu

**Affiliations:** 1 Bioproduction Research Institute, National Institute of Advanced Industrial Science and Technology (AIST), 2-17-2-1 Tsukisamu-Higashi, Toyohira-ku, Sapporo, 062-8517 Japan; 2 Cellular and Molecular Biotechnology Research Institute, National Institute of Advanced Industrial Science and Technology (AIST), Central 5, 1-1-1, Higashi, Tsukuba, Ibaraki 305-8565 Japan; 3 Graduate School of Life Science, Hokkaido University, 8, Kita 10-jo-Nishi, Kita-ku, Sapporo, 060-0810 Japan; 4 Cosmo Bio Co., Ltd., 3-513-2, Zenibako, Otaru, Hokkaido 047-0261 Japan

**Keywords:** miRNAs, Antisense oligonucleotide therapy

## Abstract

MicroRNAs (miRNAs) regulate cancer cell proliferation by binding directly to the untranslated regions of messenger RNA (mRNA). MicroRNA-148a (miR-148a) is expressed at low levels in breast cancer (BC). However, little attention has been paid to the sequestration of miR-148a. Here, we performed a knockdown of miR-148a using anti-miRNA oligonucleotides (AMOs) and investigated the effect on BC cell proliferation. BC cell proliferation was significantly suppressed by AMO flanked by interstrand cross-linked duplexes (CL-AMO), whereas single-stranded and commercially available AMOs had no effect. The suppression was caused by sequestering specifically miR-148a. Indeed, miR-148b, another member of the miR-148 family, was not affected. Importantly, the downregulation of miR-148a induced a greater and longer-lasting inhibition of BC cell proliferation than the targeting of oncogenic microRNA-21 (miR-21) did. We identified thioredoxin-interacting protein (*TXNIP*), a tumor suppressor gene, as a target of miR-148a and showed that CL-AMO provoked an increase in *TXNIP* mRNA expression. This study provide evidence that lowly expressed miRNAs such as miR-148a have an oncogenic function and might be a promising target for cancer treatment.

## Introduction

Breast cancer (BC) is the most common cancer affecting women worldwide. Chemotherapy, endocrine therapy, and targeted therapy are widely used, alone or in combination, to treat BC^1^. However, the majority of BC patients develop drug resistance due to tumor heterogeneity^2^. Therefore, new therapeutic approaches need to be explored.

One potential therapeutic target for BC is the regulation of microRNA (miRNA)^3^, which are endogenous 18–23-nucleotide small noncoding RNAs that bind to the 3′-untranslated region (3′-UTR) of specific target messenger RNAs (mRNAs). They regulate gene expression post-transcriptionally^4^ and participate in numerous cellular processes. Therefore, abnormal miRNA activity might contribute to various diseases, including BC^5^. The most typical oncogenic microRNA (oncomiR), microRNA-21 (miR-21; hsa-miR-21-5p), is overexpressed in BC tissues and cell lines, and its inhibition decreases BC cell proliferation^6–8^. Anti-microRNA oligonucleotides (AMOs) are synthetic oligonucleotides containing sequences complementary to their target miRNAs and inhibiting miRNA function^9^. The AMO performances are enhanced by various chemical modifications and/or unique secondary structures^10–16^. Advanced AMOs, flanked by interstrand cross-linked duplexes (CLDs) on both the 5′- and 3′-termini of the antisense sequences, show significantly higher inhibition activity in luciferase reporter assays than other modified AMOs do^17^. The CLD-modified AMO (CL-AMO) targeting miR-21 (CL-miR21) inhibits BC cell proliferation by regulating a target of miR-21, the endogenous phosphatase and tensin homolog deleted from chromosome 10 (*PTEN*) mRNA^8^. Altogether, these data suggest that CL-AMO is suitable for the regulation of highly expressed miRNA functions. However, their effects on lowly expressed miRNAs have not yet been investigated.

A member of the miR-148/152 family, microRNA-148a (miR-148a; hsa-miR-148a-3p), is a known tumor suppressor (anti-oncomiR). Overexpression of miR-148a inhibits cancer cell proliferation and induces apoptosis in colorectal^18^, gastric^19^, and hepatocellular cancers^20^. Similarly, in BC, miR-148a inhibits cell proliferation and migration^21^. The role of miR-148a has been evaluated exclusively by overexpression experiments in BC cells. The effect of the knockdown of miR-148a has not been fully clarified most likely because miR-148a is expressed at a low level in the cell^22–25^.

Another member of the miR-148/152 family, microRNA-148b (miR-148b; hsa-miR-148b-3p), has the same seed sequence as that of miR-148a, except for two nucleotides at the 9–10 positions from each 5′-terminus^26^. It is also an anti-oncomiR in various cancers, including BC^27–30^. However, contradictory results have shown that miR-148b is upregulated in BC patients and is a potential BC biomarker^31,32^.

The present study aims to investigate whether the knockdown of miR-148a could affect BC proliferation. We identified direct target mRNAs of miR-148a using mRNA microarray and online database analyses. Our results not only clarify the unknown functions of miR-148a in BC but also demonstrate the advantages of using CL-AMO in miRNA inhibition.

## Results

### CL-AMO-targeting miR-148a (CL-miR148a) can inhibit BC cell proliferation

**Figure 1:**
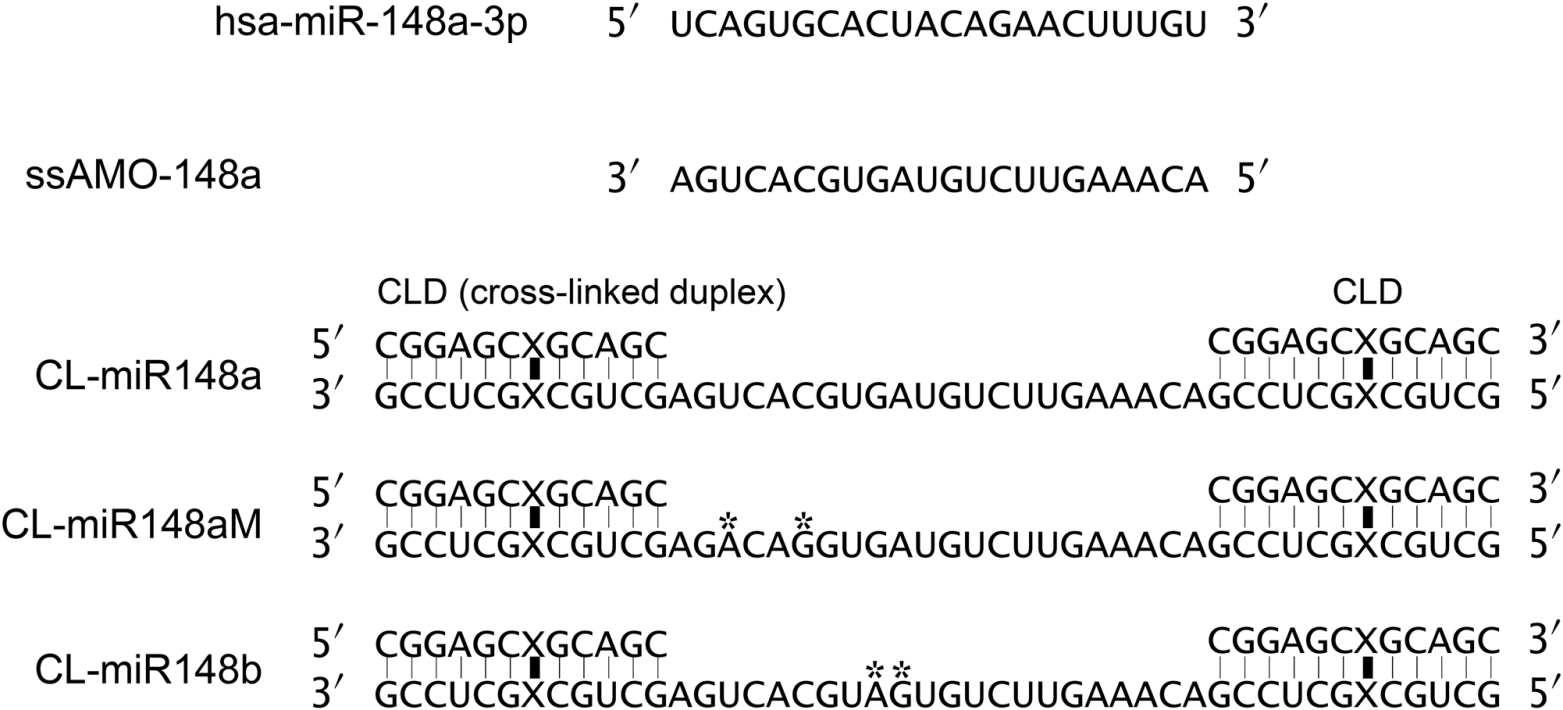
Sequences of miR-148a, ssAMO-148a, and CL-AMOs. CL-miR148a holds CLDs at both 5′- and 3′-termini of the antisense sequence complementary to miR-148a. CL-miR148aM is similar to the antisense, except that it contains two mismatched bases identified by asterisks. CL-miR148b is designed to hybridize with miR-148b. The vertical bold lines and Xs indicate cross-linker and cross-linked sites, respectively.

**Figure 2:**
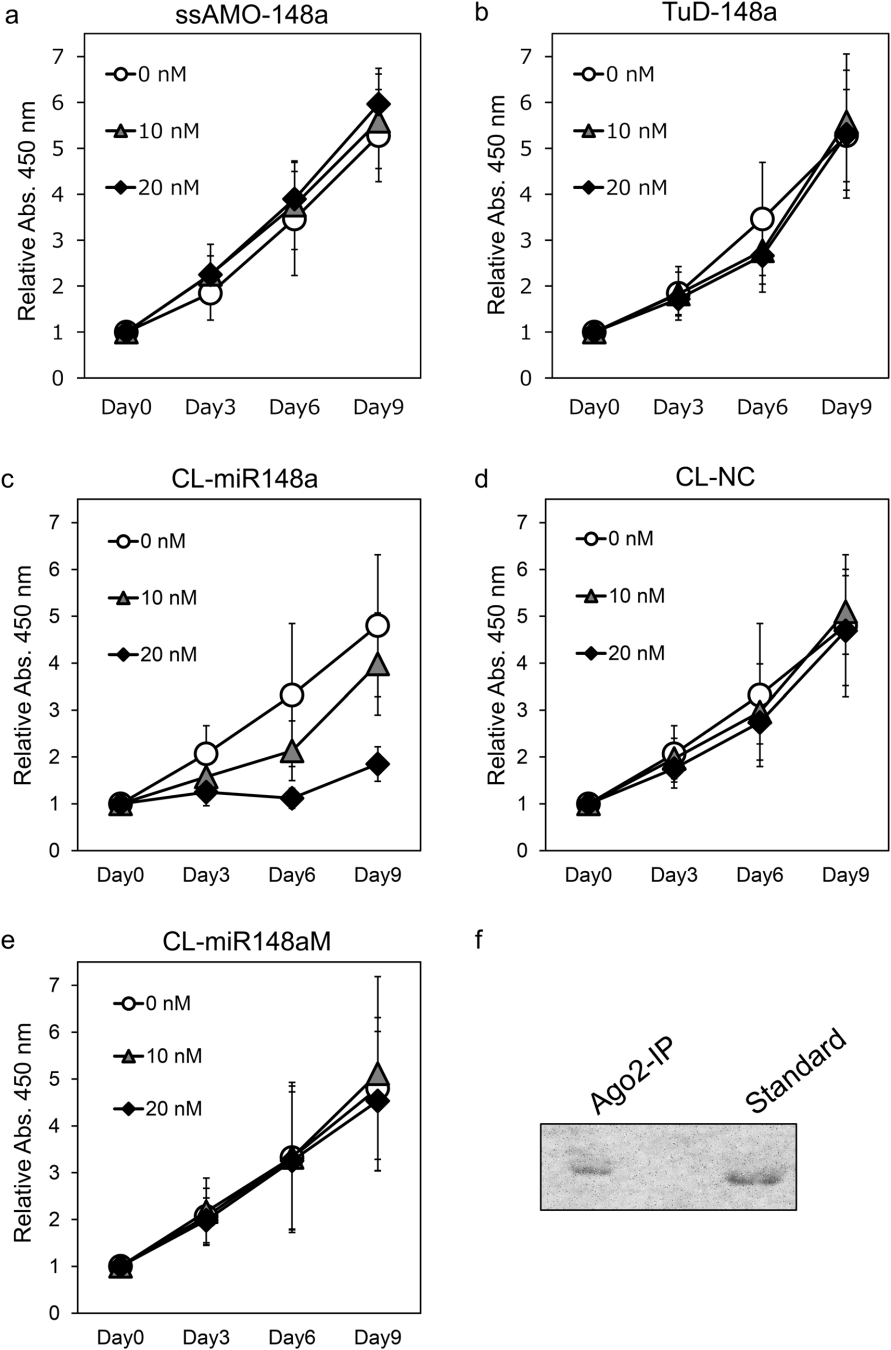
Analysis of cell proliferation after transfection with AMOs. (**a**–**e**) Plots of relative numbers of cells versus days after transfection with (**a**) ssAMO-148a, (**b**) TuD-148a, (**c**) CL-miR148a, (**d**) CL-NC, and (**e**) CL-miR148aM at a concentration of 0 (open circles), 10 (gray solid triangles), or 20 nM (black solid diamonds). Error bars represent SD. (**f**) Northern blot analysis of CL-miR148a coimmunoprecipitated with Ago2 (Ago2-IP) after transfection. “Standard” indicates CL-miR148a used for transfection. The uncropped image is shown in Supplementary Fig. S6.

We focused on miR-148a in BC and first measured miR-148a expression in MCF-7 cells. The expression of miR-148a was quite low (Supplementary Fig. S1) compared with that of miR-21, which is frequently overexpressed in cancer^33^. We used a single-stranded AMO synthesized from 2′-*O*-methyl RNAs (MeRNAs) (ssAMO-148a; Fig. 1) and commercially available AMOs to knockdown miR-148a. MCF-7 cells were transfected with each AMO independently, and the number of MCF-7 cells was counted after culture. The ssAMO-148a, which only has a complementary sequence for miR-148a (ssAMO-148a; Fig. 1), did not affect MCF-7 cell proliferation (Fig. 2a and Supplementary Fig. S2a). The tough decoy (TuD) AMO^12^-targeting miR-148a (TuD-148a) showed a slight inhibitory effect at 20 nM (Fig. 2b), but higher concentrations did not provide further inhibition (Supplementary Fig. S2b).

Next, we used a CL-AMO that had CLDs at the 5′- and 3′-ends^17,34^. The CLDs enable CL-AMO to stably hybridize with its target RNA, which allows a long-lasting action in cells^8,17^. The CL-AMO targeting anti-miR-148a (CL-miR148a) was synthesized from MeRNAs to have cross-linked 12-mer duplexes at both the 5′- and 3′-termini of the 22-mer antisense (Fig. 1 and Supplementary Fig. S3). In addition to CL-miR148a, we constructed a negative control with a scrambled sequence (CL-NC: Supplementary Fig. S4) and a mismatch AMO (CL-miR148aM; Fig. 1), containing two mismatched base pairs in the seed region, which is essential for miRNA–target mRNA binding^35,36^. Surprisingly, CL-miR148a showed a dose-dependent activity (Fig. 2c), and transfection with 20 nM CL-miR148a completely inhibited MCF-7 cell proliferation after 6 days. Transfection of CL-NC and CL-miR148aM had no significant effect (Fig. 2d,e), which confirmed that CL-miR148a antiproliferative action was mediated by its binding to miR-148a. Moreover, we performed the inhibition of miR-148a in ZR-75-1 breast cancer cell line and confirmed the antiproliferative effect of CL-miR148a in another cell line (Supplementary Fig. S5).

We also investigated the effects of miR-148b in MCF-7 cell. The transfection of the CL-AMO targeting miR-148b (CL-miR148b; Fig. 1) induced a very weak inhibition at 3 and 6 days after transfection, but the effect was lost after 9 days (Supplementary Fig. S2c). The difference between CL-miR148a and CL-miR148b activities did not result from target amounts as both miRNAs were expressed to similar extent in MCF-7 cells (Supplementary Fig. S2d). It also indicates that the inhibitory effect of CL-miR148a was not mediated by miR-148b downregulation.

We performed immunoprecipitation (IP) using anti-Ago2 antibodies after CL-miR148a transfection to investigate whether CL-miR148a binds to the miRNA-induced silencing complex (miRISC). CL-miR148a was recovered in the IP samples, confirming that CL-miR148a binds to miRISC (Fig. 2f and Supplementary Fig. S6).

**Figure 3:**
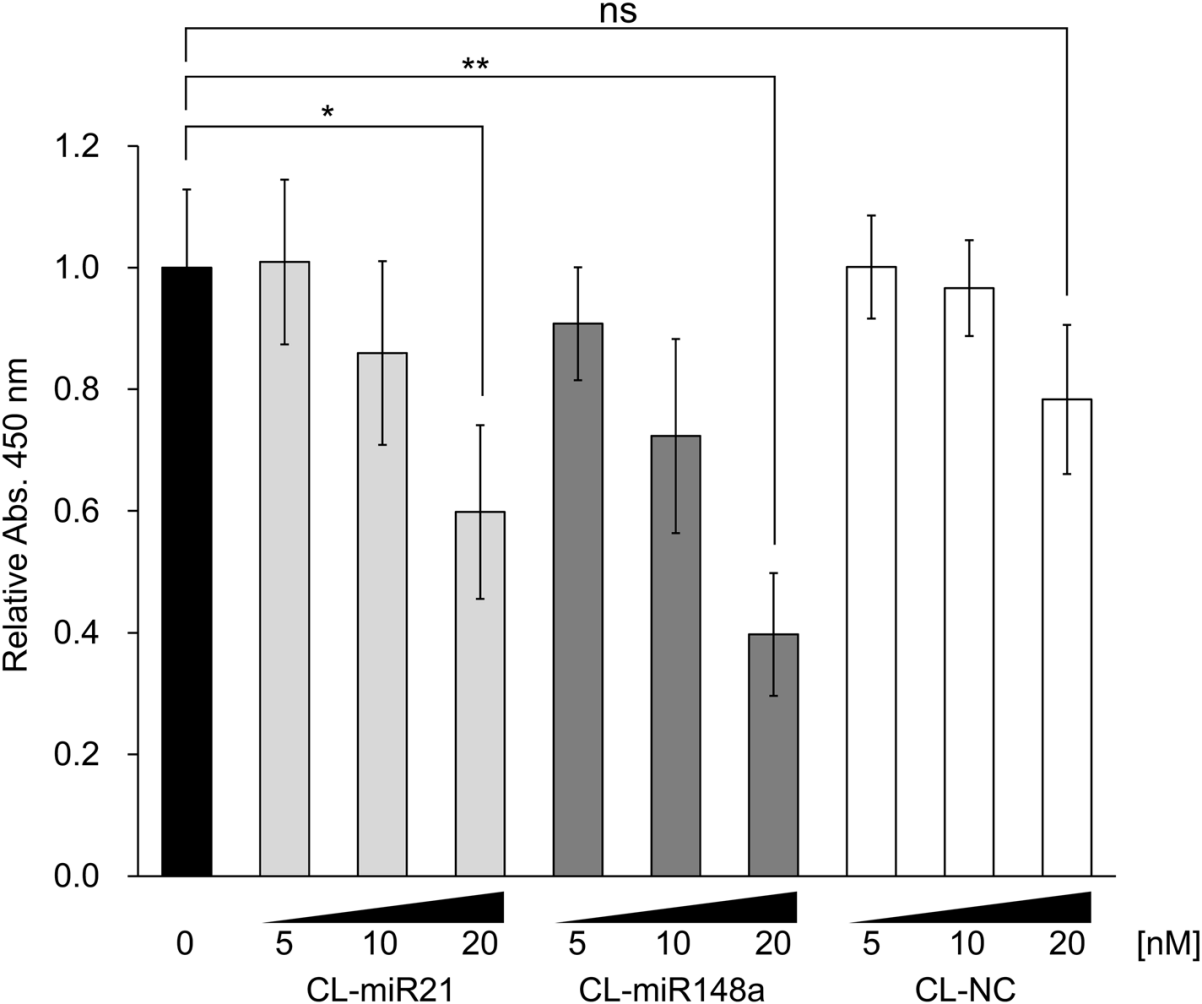
Relative number of MCF-7 cells transfected with CL-AMOs (CL-miR21, CL-miR148a, or CL-NC) 6 days after transfection. AMO concentrations varied from 0 to 20 nM, as labeled below the x-axis. Error bars represent SD. “ns” indicates insignificant; *p < 0.05; **p < 0.01.

miR-21 is one of the most well-defined oncomiRs, and the downregulation of miR-21 suppresses cell proliferation of BC cells^6,7^. We prepared a CL-AMO-targeting miR-21 (CL-miR21; Supplementary Fig. S4) and compared the inhibition activities of CL-miR21 and CL-miR148a. As previously reported, CL-miR21 inhibited cell proliferation at 10 and 20 nM 6 days after transfection (Fig. 3). Interestingly, the antiproliferative activity of CL-miR148a was greater than the CL-miR21 effect at all concentrations tested. The biggest significant difference was observed for the 20 nM samples. It is notable that the inhibitory activity of CL-miR148a was maintained even after 9 days whereas CL-miR21 did not display a suppressive effect of this duration (data not shown).

We performed quantitative polymerase chain reaction (qPCR) to measure miRNA expression level after CL-AMO transfection but did not obtain reproducible results (data not shown). This is consistent with previous results reporting that high-affinity AMOs do not mediate the degradation of miRNAs^17,37^ and sometimes directly prevent qPCR reaction^38^.

### Investigation of target genes regulated by CL-miR148a

**Figure 4:**
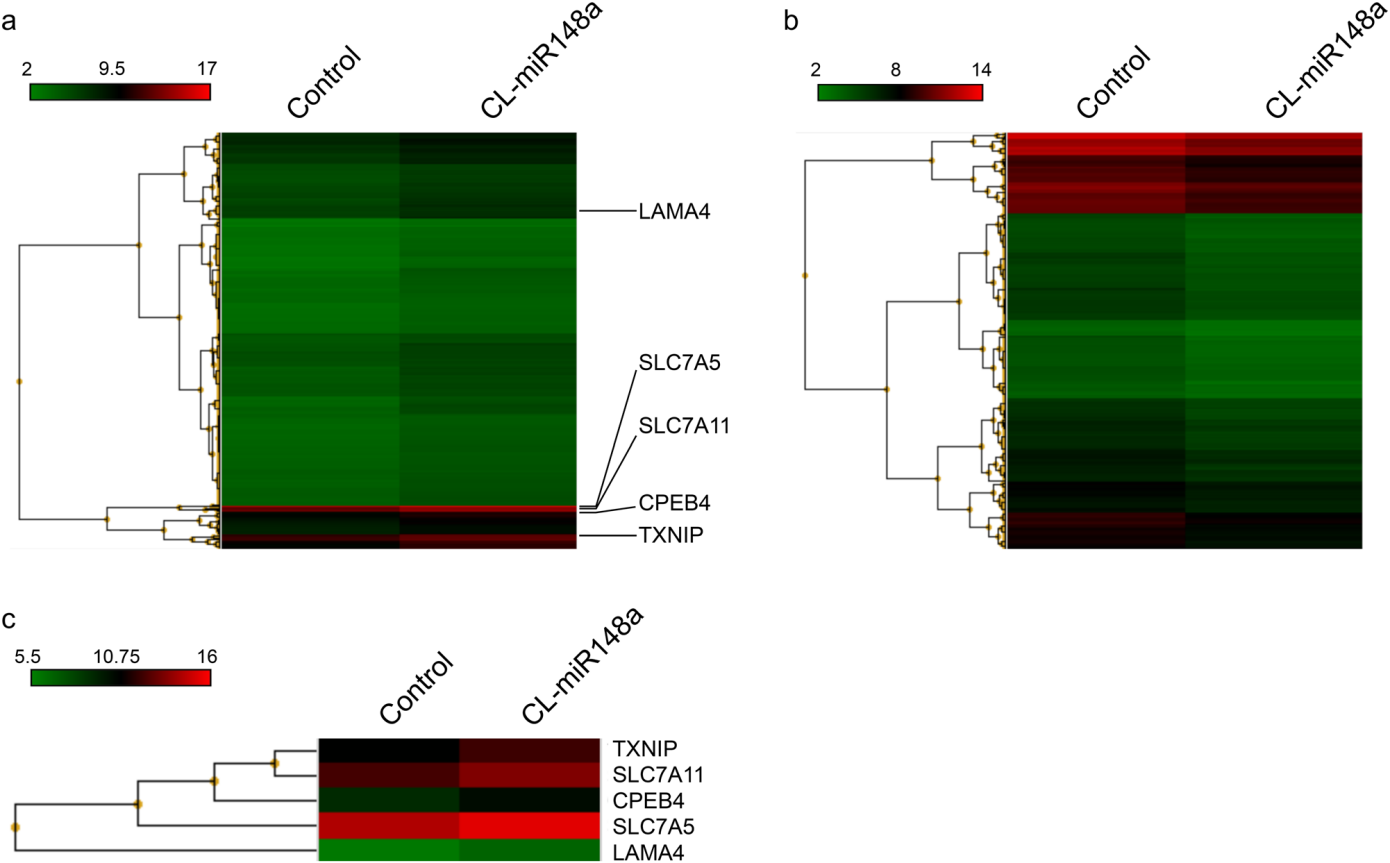
Heatmaps of the differential expression of mRNAs in no AMO (control) or CL-miR148a-transfected MCF-7 cells. High expression levels are in red, and low expression levels are in green. Compared with the control, 461 genes were upregulated by CL-miR148a transfection (**a**), whereas 462 genes were downregulated (**b**). After performing an online database analysis, five candidate genes were selected. The five candidate genes (*TXNIP*, *SLC7A11*, *CPEB4*, *SLC7A5*, and *LAMA4*) are marked on the heatmap in (**a**), which is enlarged in (**c**).

**Figure 5:**
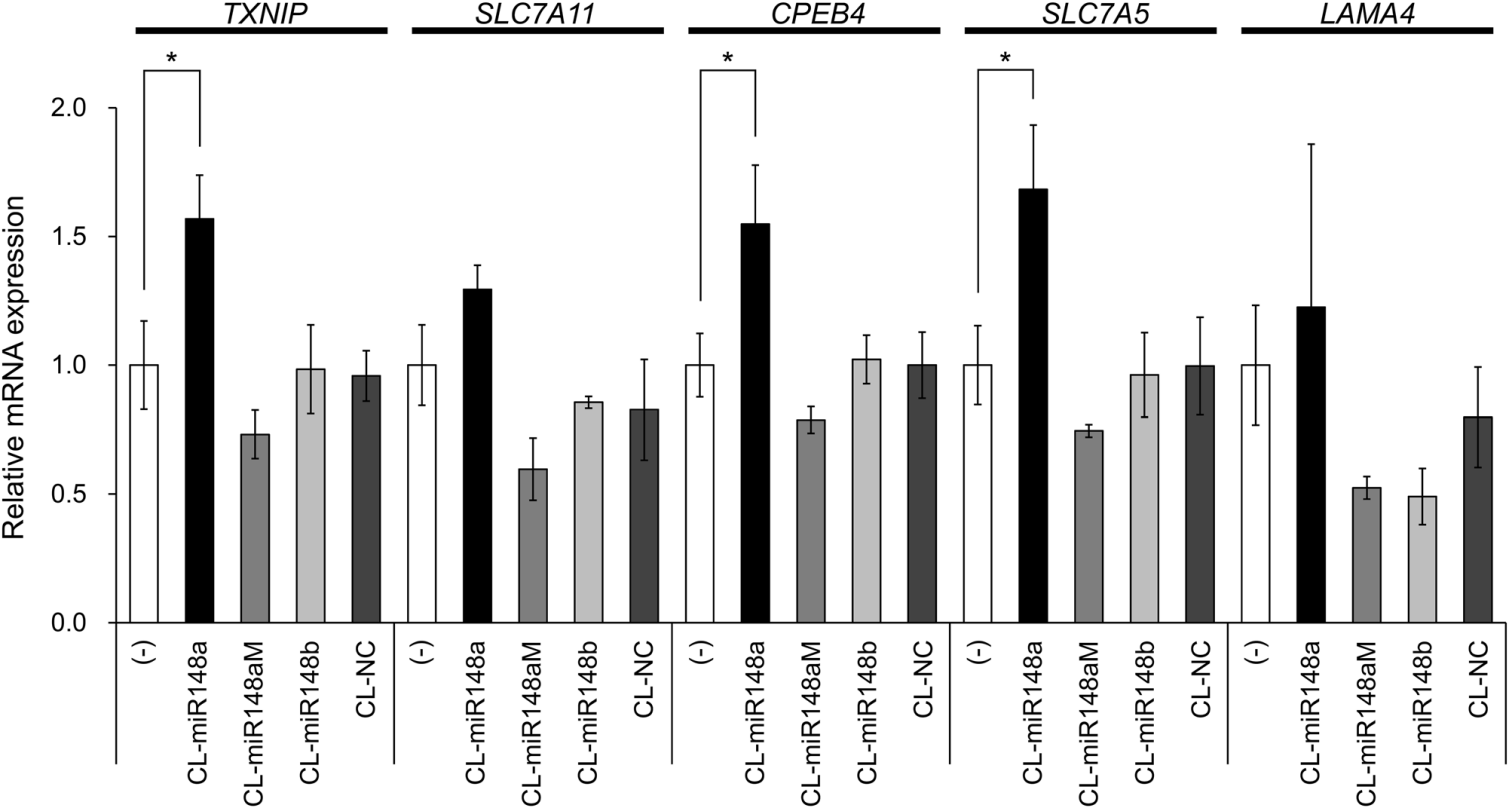
Changes in mRNA expression induced by CL-AMO transfection. The expression of the mRNA, relative to the control without AMO, was plotted against CL-AMOs. The name of each gene appears at the top of the graph, and the CL-AMO, including the control, is shown below the horizontal x-axis. Error bars represent SD; *p < 0.05.

We analyzed the gene expression profile by mRNA microarrays to identify the genes controlled by miR-148a. Genes were considered differentially expressed when more than 1.5-fold changes were measured upon CL-miR148a addition. We found 461 genes that were upregulated (Fig. 4a) and could constitute direct targets of miR-148a, and 462 genes were downregulated (Fig. 4b). An online database search using TargetScan 7.2 identified 802 genes predicted to contain binding sequences to miR-148a in the 3′-UTR of their mRNAs. Using microarray and online database analysis, we selected five candidates as direct miR-148a target: solute carrier family 7 member 11 (*SLC7A11*), thioredoxin-interacting protein (*TXNIP*), cytoplasmic polyadenylation element-binding protein 4 (*CPEB4*), solute carrier family 7 member 5 (*SLC7A5*), and laminin subunit alpha 4 (*LAMA4*) (Fig. 4c and Supplementary Fig. S7). MCF-7 cells were transfected with CL-miR148a, and qPCR analysis of *SLC7A11*, *TXNIP*, *CPEB4*, *SLC7A5*, and *LAMA4* was performed. The mRNA expression levels of *TXNIP*, *CPEB4,* and *SLC7A5* significantly increased, whereas *SLC7A11* and *LAMA4* mRNA were only slightly increased (Fig. 5). These results indicate that *TXNIP*, *CPEB4*, and *SLC7A5* were plausible candidate genes involved in the inhibition of MCF-7 cell proliferation. CL-AMOs that have no complementary sequences to miR-148a (CL-miR148aM, CL-miR148b, and CL-NC) did not affect the mRNA expression level of these genes (Fig. 5). We considered that *TXNIP* was the most promising miR-148a target gene in MCF-7 cells as it was shown to contribute to a better prognosis of BC^39^ and inhibits BC cell proliferation^40^. Recently, the direct binding between miR-148a and the 3′-UTR of *TXNIP* was reported in noncancerous cells. Moreover, an anti-miR-148a oligonucleotide was shown to upregulate TXNIP protein in hepatocytes^41^ and cardiomyocytes^42^. We also investigated whether ssAMO or TuD-AMO-targeting miR-148a upregulated *TXNIP* expression. As shown in Supplementary Fig. S8, ssAMO had no effect, whereas TuD elicited only a slight effect on *TXNIP* expression. Therefore, *TXNIP* expression might be closely associated with BC cell proliferation regulated by CL-miR148a, as discussed below.

## Discussion

Aberrant expression of miRNAs can cause various cancers, which attracted attention on miRNAs as potential therapeutic targets and biomarkers of cancer^43,44^. Inhibition of miR-21, one of the earliest identified oncomiRs^45^, represents a potential therapeutic target^33^. In BC, expression of miR-148a is low, and its overexpression inhibits BC cell proliferation and migration^21–23,25^. As miR-148a has an antioncogenic function, knockdown experiments of miR-148a in BC were not considered.

In this study, we examined the effects of miR-148a knockdown. As the expression of miR-148a is low, we took advantage of CL-AMOs, which have high binding affinity for target miRNAs and a durable action in cells, to sequester miR-148a. CL-miR148a clearly inhibited BC cell proliferation in a dose-dependent manner (Fig. 2c and Supplementary Fig. S5), and the effect lasted for 9 days after transfection. Conversely, ssAMO-148a or TuD AMOs had no significant effect even at a concentration tenfold higher than that of CL-miR-148a (Fig. 2a, b, and Supplementary Fig. S2a, b). The mismatched CL-AMO (CL-miR148aM) did not inhibit BC cell proliferation (Fig. 2e). CL-miR148a was found to be bound to the Ago2–miRNA complex by IP (Fig. 2f), which confirmed that CL-miR148a functioned by binding between CL-AMO and miR-148a.

BC cell proliferation was not affected by CL-miR148b, which was specific for miR-148b (Supplementary Fig. S2c). This excluded a possible cross-reaction between CL-miR148a and miR-148b. Thus, miR-148b, unlike miR-148a, is irrelevant for suppressing BC cell growth.

Notably, even though miR-148a had a much lower expression level than miR-21 in MCF-7 cells (Supplementary Fig. S1)^46^, the downregulation of miR-148a inhibited cell growth more efficiently than the decrease of miR-21 did (Fig. 3). This result suggests that miRNAs with low expression levels are also involved in cancer cell proliferation.

**Figure 6:**
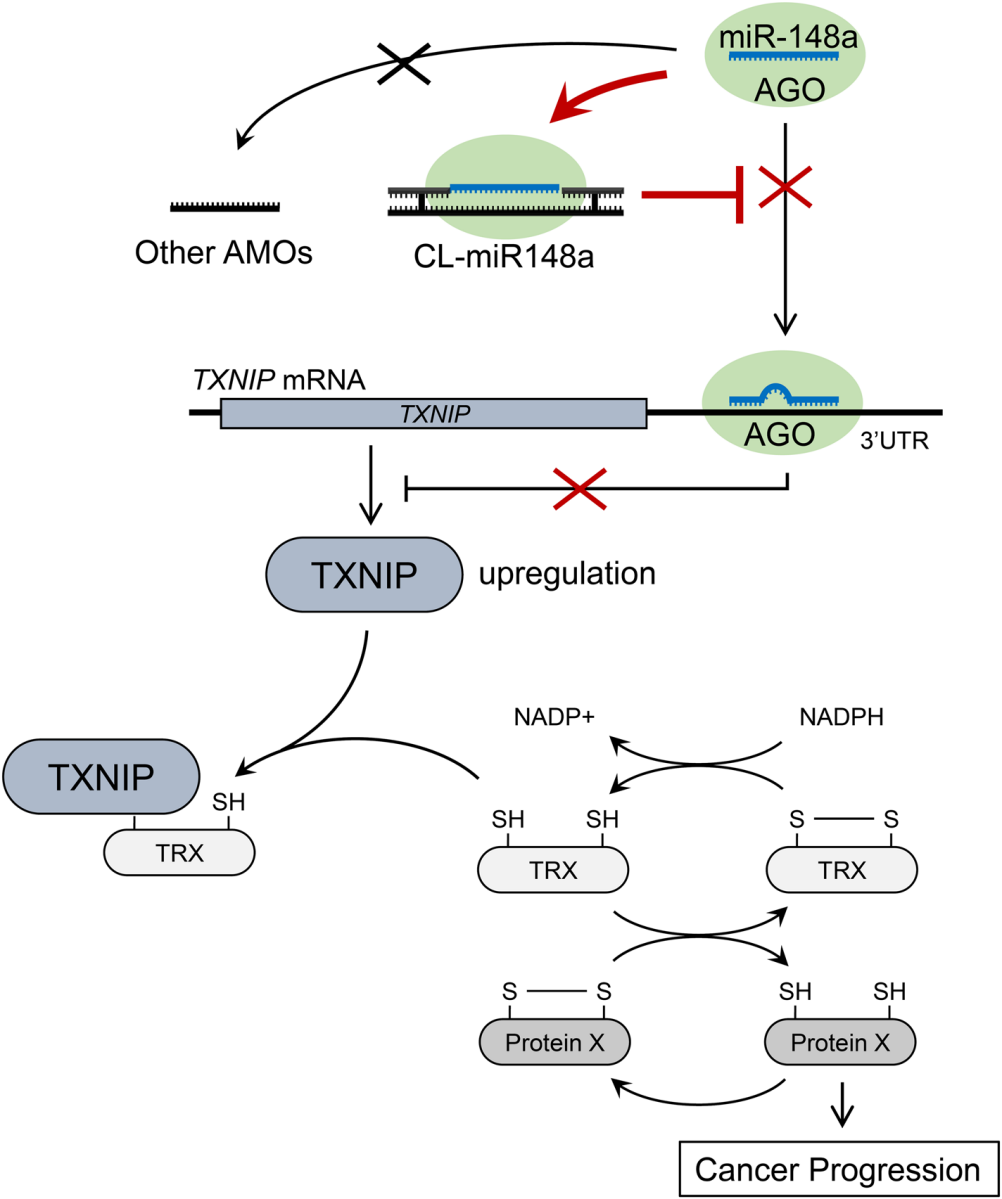
Schematic diagram of CL-miR148a-mediated inhibition of BC cell proliferation. The reduced form of TRX (thioredoxin) displays an antioxidant activity by reducing the oxidized protein X. This antioxidant function is associated with cancer progression. *TXNIP* binds to reduced TRX and prevents protein X reduction, resulting in the inhibition of tumorigenesis in BC. *TXNIP* expression is regulated by miR-148a binding to the 3′-UTR of the mRNA. CL-miR148a antagonizes miR-148a binding to *TXNIP* mRNA, resulting in the upregulation of *TXNIP* expression. By contrast, other AMOs could not stably sequester miR-148a. The red X marks the steps inhibited by the addition of AMO.

The CLD structure is able to stabilize the hybridization of adjacent single-stranded antisense. We previously reported that CL-AMOs had a much higher melting temperature when bound to target RNAs than standard single-stranded antisense RNAs had^17^. Thus, we speculate that CL-AMOs tightly bind to their targets irreversibly, resulting in the complete sequestration or degradation of the target miRNA (Fig. 6, top).

To identify the CL-miR148a target genes associated with the inhibition of MCF-7 cell proliferation, we conducted mRNA microarray analysis after CL-miR148a transfection. We considered upregulated genes as direct targets of miR-148a (Fig. 4a) and narrowed them down to five genes by referring to an online database for miR-148a target prediction (Fig. 4c and Supplementary Fig. S7). Importantly, three of the five genes were upregulated upon the addition of CL-miR148a (Fig. 5). We specifically focused on *TXNIP*, which was first identified as a 1,25-dihydroxyvitamin D_3_-inducible gene^47^, because recent studies have reported the direct binding of miR-148a to the 3′-UTR of *TXNIP* in hepatocytes^41^ and cardiomyocytes^42^. In contrast to CL-miR148a, ssAMO-148a did not affect *TXNIP*, whereas TuD-148a induced a slight dose-dependent upregulation of *TXNIP*, consistent with its effect on cell proliferation (Supplementary Fig. S8). These results suggest that inhibition of BC cell proliferation is caused by changes in the *TXNIP* expression level.

TXNIP directly binds to the reduced form of thioredoxin (TRX) (Fig. 6, middle)^48^. TRX is an antioxidant protein that reduces oxidized proteins and protects cells from oxidative stress-induced apoptosis. It was also shown to contribute to cancer progression (TRX system; Fig. 6, bottom)^49,50^. *TRX* is overexpressed in BC^51,52^, and *TRX*-transfected MCF-7 cells display a higher proliferation rate^53^. Therefore, the TRX system is closely related to MCF-7 cell growth^54^. The binding of TXNIP to TRX decreases the amounts of the TRX reduced form, resulting in the inhibition of BC tumorigenesis^39,40^. CL-miR148a transfection upregulated *TXNIP* mRNA expression (Fig. 5) but caused no change in *TRX* expression level (Supplementary Fig. S9). TXNIP inhibits TRX activity by protein–protein interaction^48^ but does not regulate *TRX* mRNA expression^55^. Therefore, it is plausible that CL-miR148a caused the inhibition of BC cell proliferation through the TXNIP and TRX system.

This is the first study reporting that miR-148a downregulation inhibits BC cell proliferation. We showed that *TXNIP* gene expression is under the control of miR-148a in MCF-7 cells (Fig. 5). CL-miR148a could reduce miR-148a to even a lower concentration in cells, resulting in the inhibition of BC cell proliferation via *TXNIP* upregulation (Fig. 6, top). These results indicate that complete inhibition of miRNA unveils unknown cellular responses, which might lead to the discovery of unknown functions of miRNAs.

Our findings provided new knowledge regarding miR-148a function in BC, and they may contribute to the development of promising strategies for BC treatment. Additionally, it showed the importance of miRNAs with low expression levels. Finally, AMO-mediated knockdown of miRNAs can provide important insights into the functions of these miRNAs.

## Methods

### AMOs

The anti-miR-148a tough decoy RNA was purchased from GeneDesign Inc. (Osaka, Japan). 22-mer single-stranded anti-hsa-miR-148a-3p and all CL-AMOs (CL-miR148a, CL-miR148aM, and CL-miR148b) were chemically synthesized from 2′-*O*-methyl RNA (MeRNA) (GeneDesign Inc.). CL-AMOs were prepared according to previous reports^17^ with slight modifications (see scheme in Supplementary Fig. S3). The molecular weights of CL-miR148a, CL-miR148b, and CL-miR148aM were confirmed by liquid chromatography–mass spectrometry (LC–MS). The ESI–MS data of all CL-AMOs are shown below.

- CL-miR148a, calculated 23,392.25, found 23,396.57;
- CL-miR148b, calculated 23,392.25, found 23,399.72;
- CL-miR148aM, calculated 23,456.03, found 23,459.85.

### Cells

We obtained the human breast cancer cell lines MCF-7 and ZR-75-1 from the American Type Culture Collection (ATCC, Manassas, VA, USA). MCF-7 cells were cultured in Dulbecco’s modified Eagle medium (DMEM; Cosmo Bio, Tokyo, Japan) supplemented with 10% fetal bovine serum (FBS; Biological Industries, Beit Haemek, Israel). ZR-75-1 cells were cultured in Roswell Park Memorial Institute (RPMI)-1640 medium (Thermo Fisher Scientific, Waltham, MA, USA) supplemented with 10% FBS. The cells were incubated in a humidified 5% CO_2_ atmosphere at 37 °C.

### Cell proliferation assay

The cells were seeded onto a 96-well plate at a density of 2 × 10^3^ cells/well (MCF-7) or 1 × 10^3^ cells/well (ZR-75-1) and allowed to adhere overnight. Then, AMOs were transfected into the cells at final concentrations of 5, 10, and 20 nM using Lipofectamine 3000 (Thermo Fisher Scientific) according to the manufacturer’s instructions. After 3 and 6 days of transfection, we replaced the cell culture medium with fresh medium. The number of cells was assessed on day 0 (day of transfection) and on days 3, 6, and 9 post-transfection using the Cell Counting Kit-8 assay (CCK-8; Dojindo, Kumamoto, Japan) according to the manufacturer’s instructions. Briefly, a CCK-8 solution containing WST-8 dye was added to the cell culture medium, and the absorbance at 450 nm was measured 90 min post-incubation using a microplate reader (TECAN, Mannëdorf, Switzerland). The cell number was expressed relatively to the value for no AMO control (single-point analysis at 6 days post-transfection) or for the day of transfection (day0) (time-course analysis for 9 days).

### Quantitative analysis of miRNA expression

Endogenous miR-148a and miR-148b expression in MCF-7 cells was analyzed as previously described^8^ using the TaqMan MicroRNA Assay (Thermo Fisher Scientific) according to the manufacturer’s instructions. U6 RNA was used as an endogenous control to normalize the relative quantitation of target miRNAs.

### Immunoprecipitation of Ago2 complex and detection of CL-miR148a

Briefly, we seeded 2.8 × 10^6^ MCF-7 cells separately in three 75 cm^2^ flasks and transfected them with CL-miR148a at a final concentration of 2 nM. We harvested the cells 24 h post-transfection and performed IP of the Ago2 complex using the microRNA Isolation Kit, Human Ago2 (Wako, Osaka, Japan), according to the manufacturer’s instructions. After ethanol precipitation, we merged the IP samples from the three flasks into one sample. Northern blot analysis of CL-miR148a was performed as previously described^17^. For CL-miR148a detection, we prepared a digoxigenin (DIG)-tailed deoxyoligonucleotide probe for the miR-148a sequence using the DIG Oligonucleotide Tailing Kit, 2nd Generation (Roche, Mannheim, Germany), and subjected it to hybridization with CL-miR148a on the nylon membrane overnight.

### mRNA microarray

MCF-7 cells mock-transfected or transfected with CL-miR148a at a final concentration of 20 nM were harvested using TRI Reagent 3 days post-transfection. Purification of mRNA and mRNA microarray analysis were performed at Macrogen Japan (Tokyo, Japan) using the human Clariom S Assay (Thermo Fisher Scientific). The data were analyzed using Affymetrix GeneChip Command Console software (Thermo Fisher Scientific), and we compared differences in gene expression between mock-transfected and CL-miR148a-transfected samples.

### Quantitative analysis of mRNA expression

MCF-7 cells were seeded onto a 48-well plate at a density of 4 × 10^3^ cells/well and transfected with AMOs, as described above, at a final concentration of 20 or 50 nM. After 3 days, the cells were harvested, merging three wells into one sample, using the RNeasy Plus Mini Kit (QIAGEN, Hilden, Germany). We purified total RNAs and synthesized cDNA as previously described^8^. Then, we performed qPCR according to a previous report^8^ using *glyceraldehyde 3-phosphate dehydrogenase* (*GAPDH*) expression to normalize the target gene expression. We calculated the expression level of target genes as 2^−ΔCt^, where ΔCt = Ct(target) − Ct(*GAPDH*).

The following primers were used for PCR:

- GAPDH forward: 5′-TTGCCCTCAACGACCACTTT-3′
- GAPDH reverse: 5′-TGGTCCAGGGGTCTTACTCC-3′
- SLC7A11 forward: 5′-GGTTATTCTATGTTGCGTCTC-3′
- SLC7A11 reverse: 5′-AATAACAGCTGGTAGAGGAG-3′
- TXNIP forward: 5′-CTGATCTATGTTAGCGTTCC-3′
- TXNIP reverse: 5′-TATCAGGGATGTTCAGATCTAC-3′
- CPEB4 forward: 5′-GAGTTGCGTTCTCTAATCAAC-3′
- CPEB4 reverse: 5′-ACCCGTTTATCTATCTCTCC-3′
- SLC7A5 forward: 5′-TCCAGATCGGGAAGGGTGAT-3′
- SLC7A5 reverse: 5′-CAGGGGCAGGTTTCTGTAGG-3′
- LAMA4 forward: 5′-GAAATTGCATTTGAAGTCCG-3′
- LAMA4 reverse: 5′-ACCTGTCCATTTTTCATGTG-3′
- TRX forward: 5′-TGGTGAAGCAGATCGAGAGC-3′
- TRX reverse: 5′-CATTTTGCAAGGCCCACACC-3′.

## Supplementary Information

Supplementary Information.
