# Supplementary material for: Stable duplex-linked antisense targeting miR-148a inhibits breast cancer cell proliferation
Source: Sci Rep. 2021 Jun 1;11:11467. doi: 10.1038/s41598-021-90972-3 (PMC8169724; doi:10.1038/s41598-021-90972-3)
Supplement: Supplementary file 1 — Supplementary Information. [file 41598_2021_90972_MOESM1_ESM.pdf]

Supplementary information

Stable duplex-linked antisense targeting miR-148a inhibits breast cancer cell proliferation

Sho Okumura, Yu Hirano and Yasuo Komatsu*

Contents

1. **Endogenous miR-21 and miR-148a expression in MCF-7 cells**
2. **Effect of high concentration of commercially available AMOs and CL-AMO targeting miR-148b on MCF-7 proliferation**
3. **Preparing CL-AMO by cross-linking 12- and 46-mer MeRNA**
4. **Sequence of CL-NC and CL-miR21**
5. **Effect of CL-miR148a on ZR-75-1 cell proliferation**
6. **Uncropped image of northern blot analysis**
7. **Target sequences of miR-148a in the 3'UTR of candidate genes**
8. ***TXNIP* mRNA expression after transfection of various AMOs**
9. ***TRX* mRNA expression after transfection of AMOs**

1. Endogenous miR-21 and miR-148a expression in MCF-7 cells

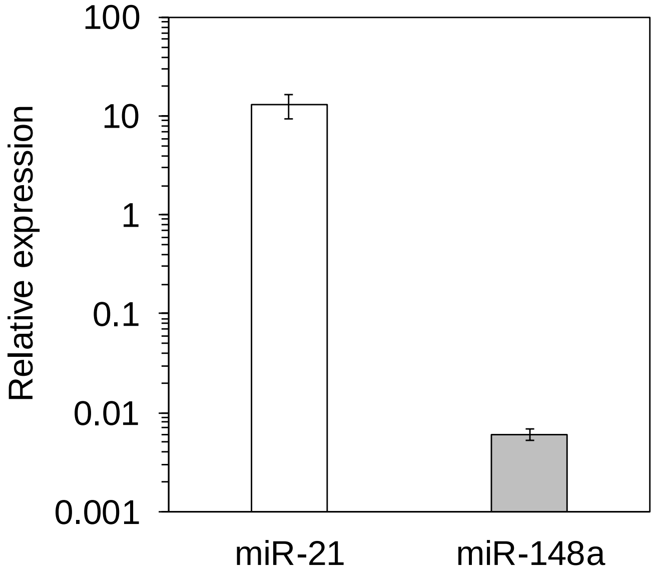

Supplementary Figure S1

qPCR analysis of miR-21 and miR-148a expression in MCF-7 cells. The expression level is indicated as a value relative to the U6 RNA expression. Error bars represent SD. Notice that the vertical axis is logarithmic.

2. **Effect of high concentration of commercially available AMOs and CL-AMO targeting miR-148b on MCF-7 proliferation**

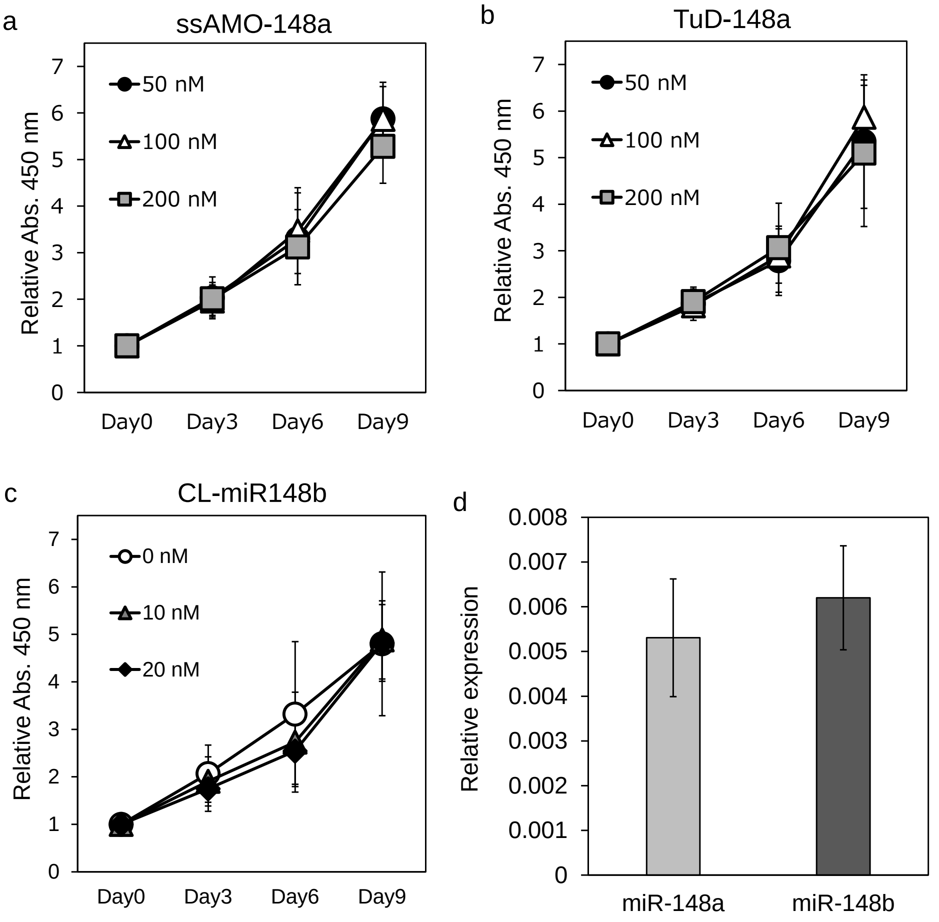

Supplementary Figure S2

Analysis of cell proliferation after transfection with AMOs. (**a–c**) Plots of relative numbers of cells versus days after transfection. (**a**) ssAMO-148a and (**b**) TuD-148a were used at a concentration of 50 (black solid circles),100 (open triangles), or 200 nM (gray solid squares). (**c**) CL-miR148b were used at a concentration of 0 (open circles), 10 (gray solid triangles), or 20 nM (black solid diamonds). Error bars represent SD. (**d**) Comparison of endogenous miR-148a and miR-148b expression in MCF-7 cells. The expression level is indicated relative to U6 RNA expression. Error bars represent SD.

3. Preparing CL-AMO by cross-linking 12- and 46-mer MeRNA

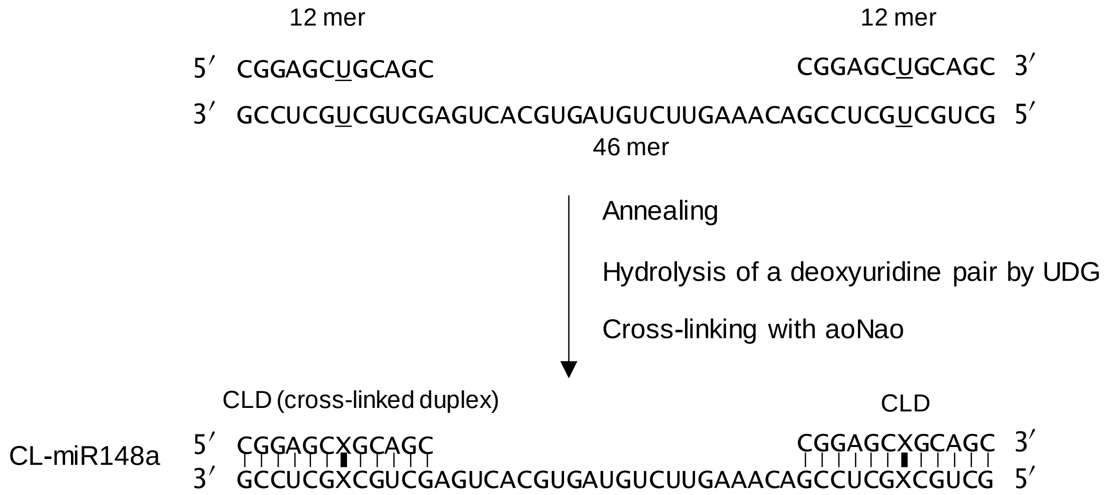

Supplementary Figure S3

CL-miR148a was constructed from cross-linking between 12- and 46-mer-containing antisense sequences of miR-148a. Both 12- and 46-mer oligonucleotides were synthesized from 2′-*O*-methyl RNA (MeRNA). Briefly, MeRNA was annealed in 197.5-μL uracil DNA glycosylase (UDG) reaction solution. Next, we added 2.5 μL of 5 units/μL of UDG to the solution and incubated it for 2.5 h at 37°C. The reaction solution was then cooled at 4°C and mixed with 20 μL of 2 mM *N*,*N*′-(naphthalene-1,5-diyl)bis[2-(aminooxy)acetamide]; aoNao) as a cross-linker. Cross-linking was performed overnight at 17°C, and the reaction product was purified using reverse-phase high-performance liquid chromatography (RP-HPLC). The other CL-AMOs (CL-miR148aM and CL-miR148b) were also prepared in the same procedure using 46-mer MeRNA containing antisense sequences of miR-148a with mismatch and miR-148b, respectively.

4. Sequence of CL-NC and CL-miR21

CL-NC

5′ CGGAGCXGCAGC CGGAGCXGCAGC 3′
3′ GCCUCGXCGUCGAGCAAUUAGCCGAUAUUAUGCGGCCUCGXCGUCG 5′

CL-miR21

5′ CGGAGCXGCAGC CGGAGCXGCAGC 3′
3′ GCCUCGXCGUCGAUCGAAUAGUCUGACUACAACUGCCUCGXCGUCG 5′

Supplementary Figure S4

CL-NC has the CLDs at both 5′- and 3′-termini of a scrambled sequence, whereas CL-miR21 has the antisense sequence complementary to miR-21. The vertical bold lines and Xs indicate cross-linker and cross-linked sites, respectively.

5. Effect of CL-miR148a on ZR-75-1 cell proliferation

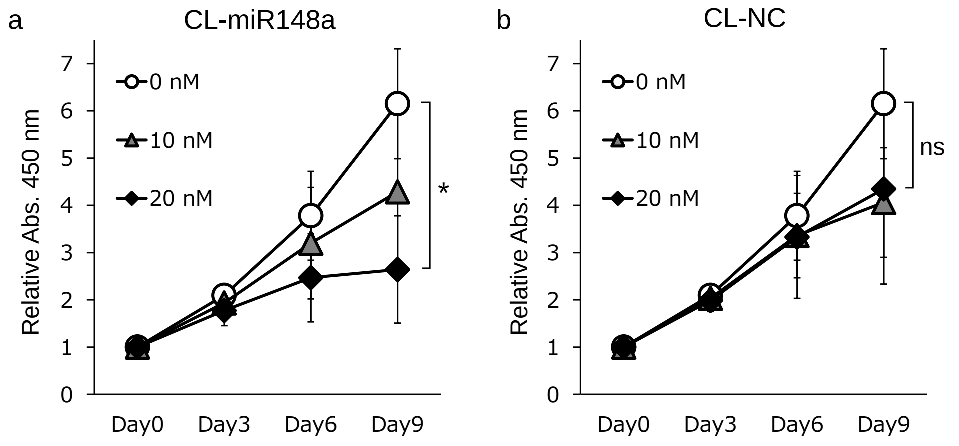

Supplementary Figure S5

Analysis of ZR-75-1 cell proliferation after transfection with AMOs. Plots of relative numbers of cells versus days after transfection with (**a**) CL-miR148a and (**b**) CL-NC at a concentration of 0 (open circles), 10 (gray solid triangles), or 20 nM (black solid diamonds). Error bars represent SD. “ns” indicates insignificant; *$p < 0.05$.

6. Uncropped image of northern blot analysis

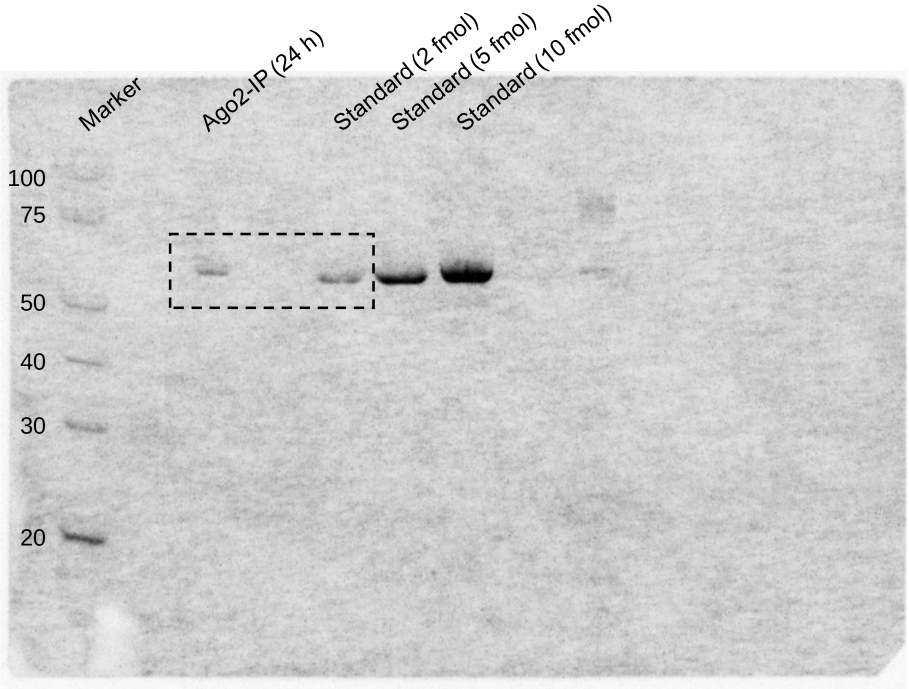

Supplementary Figure S6

Northern blot analysis of CL-miR148a co-immunoprecipitated with Ago2 (Ago2-IP) after transfection. Standard indicates CL-miR148a containing 2, 5 or 10 fmol per lane. The region of interest in this image used to Figure 2f is shown with the dotted squares.

7. Target sequences of miR-148a in the 3’UTR of candidate genes

TXNIP 3’UTR

Position 702-708

miR-148a

```
5'  ...AUUUUUGGAGCCUAUUGCACUGU...  3'
             |     || |||||||
     3' UGUUUCAAGACAUCACGUGACU  5'
```

Position 1220-1226

```
5'  ...CUGUCCUGUGUCAGAGCACUGAG...  3'
             |     || |||||||
    3' UGUUUCAAGACAUCACGUGACU  5'
```

SLC7A11 3’UTR

Position 6673-6679

```
5'  ...AUUACAUGGUAGUGAUGCACUGG...  3'
          | |     ||| |||||||
     3' UGUUUCAAGACAUCACGUGACU  5'
```

Position 7467-7473

```
5'  ... UUCUUAGGGUUCCUA---GCACUGAU...  3'
             |  ||||      |||||||
        3' UGUUUCAAGACAUCACGUGACU  5'
```

CPEB4 3’UTR

Position 1214-1220

```
5'  ...AGUUGCAAAGUGUUU---UGCACUGU...  3'
            ||||||  |    |||||||
        3' UGUUUCAAGACAUCACGUGACU  5'
```

Position 2384-2390

```
5'  ...AAAUUAUGAAUGUCGUGCACUGG...  3'
        |   |    | | ||||||||
     3' UGUUUCAAGACAUCACGUGACU  5'
```

SLC7A5 3’UTR

Position 446-453

```
5'  ...UUGCUACCACAGACUUGCACUGA...  3'
            |   | |   |||||||
     3' UGUUUCAAGACAUCACGUGACU  5'
```

LAMA4 3’UTR

Position 41-47

```
5'  ... ACAAAGUUCUUUAGAGCACUGAA...  3'
        |||||||||| ||| |||||||
     3' UGUUUCAAGACAUCACGUGACU  5'
```

Supplementary Figure S7

Targets prediction of miR-148a binding sites in the 3′UTR of the five candidate genes. The miR-148a sequence is indicated in bold characters and vertical lines of each complex show Watson–Crick base pairs. Two binding sites are predicted for *TXNIP*, *SLC7A11*, and *CPEB4* and a single binding site for *SLC7A5* and *LAMA4*.

8. *TXNIP* mRNA expression after transfection of various AMOs

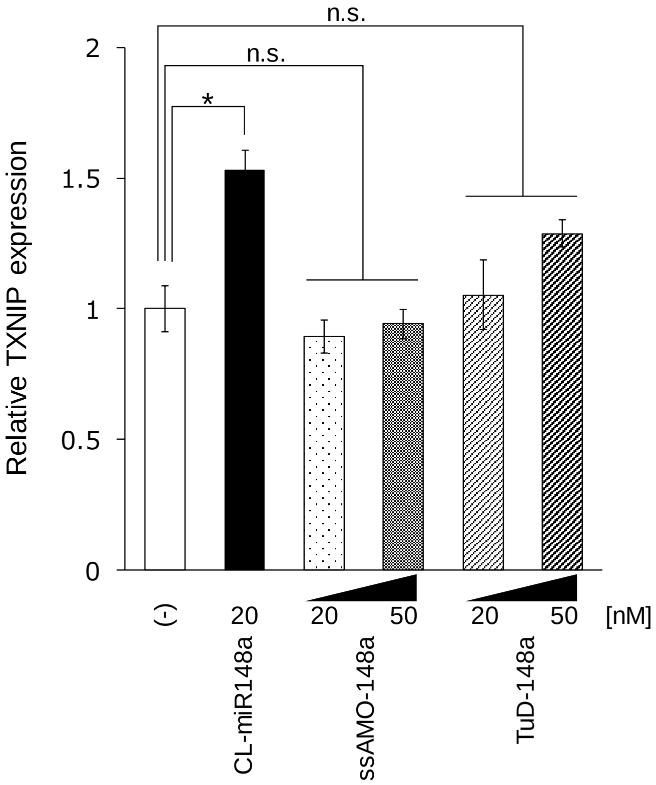

Supplementary Figure S8

Changes in *TXNIP* mRNA expression induced by various AMO transfections. The mRNA expression values relative to the control without AMO were plotted against AMOs. CL-miR148a was transfected at 20 nM, whereas ssAMO-148a and TuD-148a were 20 nM and 50 nM, as shown below the horizontal x-axis. Error bars represent SD. “ns” indicates insignificant; *$p < 0.05$.

9. *TRX* mRNA expression after transfection of AMOs

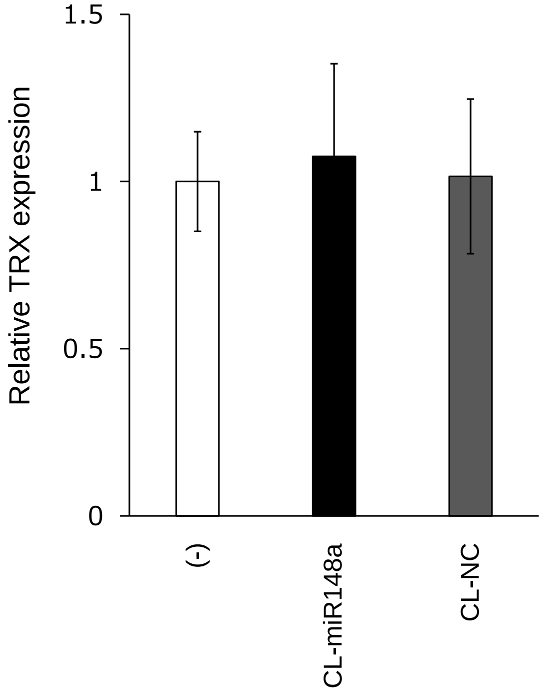

Supplementary Figure S9

TRX mRNA expression level after AMO transfections. The mRNA expression values relative to the control without AMO were plotted against CL-AMOs. Error bars represent SD.
